# Treatment of Fatigue in Multiple Sclerosis Patients: A Neurocognitive Approach

**DOI:** 10.1155/2011/670537

**Published:** 2011-09-08

**Authors:** Mauro Catalan, Alessandra De Michiel, Alessio Bratina, Susanna Mezzarobba, Lorella Pellegrini, Roberto Marcovich, Francesca Tamiozzo, Giovanna Servillo, Laura Zugna, Antonio Bosco, Arianna Sartori, Gilberto Pizzolato, Marino Zorzon

**Affiliations:** ^1^Department of Medical, Surgical and Health Sciences, Cattinara Hospital, University of Trieste, Strada di Fiume 447, 34149 Trieste, Italy; ^2^School of Physiotherapy, University of Trieste, Via G. Pascoli 31, 34129 Trieste, Italy

## Abstract

The objective of the study was to treat fatigue in patients with multiple sclerosis (MS) by a neurocognitive rehabilitation program aimed at improving motor planning by using motor imagery (MI). Twenty patients with clinically definite MS complaining of fatigue were treated for five weeks with exercises of neurocognitive rehabilitation twice a week. Patients were evaluated by Fatigue Severity Scale (FSS), Modified Fatigue Impact Scale (MFIS), MSQoL54, Expanded Disability Status Scale (EDSS), and MS Functional Composite (MSFC). After treatment, a decrease in fatigue was detected with both FSS (*P* = 0.0001) and MFIS (*P* = 0.0001). MSFC (*P* = 0.035) and MSQoL54 (*P* = 0.002) scores improved compared to baseline. At six-month followup, the improvement was confirmed for fatigue (FSS, *P* = 0.0001; MFIS *P* = 0.01) and for the physical subscale of MSQoL54 (*P* = 0.049). No differences in disability scales were found. These results show that neurocognitive rehabilitation, based on MI, could be a strategy to treat fatigue in MS patients.

## 1. Introduction

Fatigue is one of the most common and disabling symptoms of multiple sclerosis (MS), affecting up to 70% of patients [1]. It is often present in the earliest phase of the disease and it is described as the worst symptom by 40–50 % of patients [2]. Though there is still no accepted definition, fatigue in neurological disorders is defined by Chaudhuri and Behan as “a difficulty in initiation of or sustaining voluntary activities” [3]. Fatigue is a multidimensional symptom since both physical and cognitive aspects are present. In MS, fatigue is primary or secondary to other conditions such as sleep disturbances, depression, or the use of immunomodulating therapies [4]. The pathogenesis of primary fatigue in MS is still unclear, but recent evidences from electrophysiological and neuroimaging studies supported the hypothesis of a central origin. Using positron emission tomography (PET), Roelcke et al. showed reduced glucose metabolism in the frontal lobe and basal ganglia in MS patients with fatigue [5]. More recently, proton magnetic resonance spectroscopy imaging revealed widespread axonal damage in MS patients with fatigue [6]. Electrophysiological studies [7, 8] gathered additional evidence for frontal lobe cortical dysfunction in MS patients complaining of fatigue. In a review, Chaudhuri and Behan hypothesized that central fatigue may be caused by a failure in the integration of the limbic input and the motor functions within the basal ganglia, thus affecting the striatal-thalamic-frontal cortical system [9]. This hypothesis has been supported by further studies which suggested an association between fatigue and damage in the basal ganglia or thalamus [10]. Indeed, a functional magnetic resonance imaging (fMRI) study demonstrated an altered recruitment of the brain sensorimotor network (including the thalamus, the cerebellum, the frontal lobe, and the cingulum) in MS patients complaining of fatigue [11]. Although these studies suggest that fatigue is related to underlying MS pathology such as demyelization or axonal loss, other possible causes, such as temperature and immune factors, may play an important role in the genesis of fatigue [12].

Fatigue management is very difficult. Many drugs, that is, amantadine, modafinil, and aminopyridines, have been used, but no pharmacological treatment proved to be effective [13–15]. Also nonpharmacological therapies, based on physical exercise, behavioural, nutritional, and physiological training, have been tested [16, 17]. Among them, aerobic exercise, yoga and cooling therapy showed a favourable effect [18–21]. Some studies focusing on rehabilitation in MS demonstrated a transitory positive effect in reducing fatigue symptoms [22], but other studies, which tested the efficacy of different specific rehabilitation programs on fatigue as compared to placebo, did not show any differences [23, 24]. 

Considering as the origin of fatigue a dysfunction of the circuits connecting the thalamus, the basal ganglia, and the frontal cortex which are involved in motor planning and execution, we hypothesized that this symptom could be reduced by a specific neurocognitive rehabilitation program, based on the neurocognitive theory of rehabilitation (NTR) and aimed at improving motor planning by using motor imagery (MI) [25]. 

MI has already been used in the rehabilitation of central nervous system (CNS) diseases, especially, as a new approach, in stroke patients. Sharma et al. suggested that MI training might have an encouraging effect on motor function after stroke, although the interpretation of the results was limited by the small sample size and the heterogeneity of subjects' characteristics [26, 27]. 

The objective of this study was to test the efficacy of such a treatment in a sample of MS patients suffering from fatigue.

## 2. Patients and Methods

### 2.1. Patients

Twenty patients with clinically definite MS [28] (18 women, 2 men; mean age 42.2 (SD 9.7) years, disease duration 6.9 (SD 4.5) years; median Expanded Disability Status Scale (EDSS) [29] score 2.0 (range 1.0–6.0; mean 2.45 (SD 1.29)) complaining of fatigue were enrolled. Sixteen patients had a relapsing-remitting (RR) disease course, three had a secondary progressive course (SP), and one had a primary progressive (PP) course. Patients with dementia (Mini Mental Status Scale [30] <24) or major depression (Beck's Depression Inventory Scale [31] >16) were excluded, as well as patients who underwent concomitant therapy with antidepressant, psychoactive, steroid, or intravenous immunosuppressive drugs. Eighteen patients were taking disease modifying therapy (interferon beta 15, glatiramer acetate 2, and azathioprine 1) while two patients had no therapy. 

At baseline, the patients were enrolled by a neurologist who assessed clinical disability, fatigue, and quality of life. Fatigue was evaluated according to the Fatigue Severity Scale (FSS) [32] and an Italian version of the Modified Fatigue Impact Scale (MFIS) [33], which is divided into physical (pMFIS), cognitive (cMFIS), and psychosocial (psMFIS) subscales. MSQoL54, divided into physical and mental health composite score (PHCS and MHCS) [34], was used to assess quality of life, while disability was evaluated by using the EDSS [29] and MS Functional Composite (MSFC) which consists of three components: 25-Foot Walking Test (25FWT), Nine-Hole Peg Test (NHPT), and Paced Auditory Serial Addition Test (PASAT) [35]. 

The study was approved by the local ethical committee and all patients gave their written informed consent. 

### 2.2. Treatment Description

All patients were treated for five weeks with two sessions per week of a rehabilitation treatment based on the neurocognitive theory of rehabilitation (NTR), for a total of ten sessions. The basic hypothesis of NTR is that, through the correct activation of the patient's cognitive processes, such as attention, memory, language, and MI, the CNS could improve movement also in pathological conditions [25, 36]. The treatment program consisted in exercises, whose aim was to modify wrong strategies in motor planning using MI. Before motor execution, kinaesthetic information was collected and processed to help planning and controlling movement. Attention and perception are fundamental functions for the integration of these processes. During the exercises the physiotherapist gave to the patients a cognitive/motor problem to be solved through the movement of body segments, performed with the physiotherapist help. Each exercise focused on one subfunction (i.e., shifting weight on one limb, raising an arm) of a complex movement (i.e., walking, combing one's hair). The patient was asked to select, without sight control, the most important complex kinaesthetic information generated by that particular movement. Then, by using the collected information, the subject was guided to create self-generated MI [37], in the attempt to modify the movement representation before its execution. The correct execution of MI was checked by temporal coupling and patient's verbal description. Finally, the movement was experienced by the patient himself, comparing imagined to actual movement, in order to consciously detect wrong motor strategies. The solution of the problem is therefore strictly connected to the correct creation of MI, the collection and interpretation of kinaesthetic information, and the proper execution of the movement. 

Along the period of training, each patient was followed by the same expert physiotherapist on a “one-to-one” basis. During the study, patients did not undergo any other rehabilitation treatment. 

### 2.3. Followup

At the end of the five weeks of treatment (first followup) and six months thereafter (second followup), a neurologist reevaluated the patient with the same methods used at baseline. Patients' level of satisfaction about treatment was also registered. Score changes between baseline and first and second followup were compared using a nonparametric Wilcoxon's test for paired samples.

## 3. Results

### 3.1. First Follow-Up Evaluation

All patients completed the programmed treatment. Clinical variable scores before and after five weeks of treatment are shown in Table 1. No differences between baseline and first follow-up EDSS scores were found. All patients reported a subjective improvement of their symptoms after treatment. A significant decrease in fatigue scale scores was detected both with FSS (*P* = 0.0001) and MFIS (*P* = 00001). Considering the MFIS subscales, significant differences were found for pMFIS (*P* = 0.0001) and cMFIS (*P* = 0.0001) but not for the psMFIS (*P* = 0.133).

The MSFC score was significantly improved compared to baseline (*P* = 0.035). Taking into consideration separately the three components of MSFC, the differences showed only trends but did not reach statistical significance. Quality of life significantly improved, since both PHCS (*P* = 0.002) and MHCS (*P* = 0.004) scores increased after treatment. 

### 3.2. Second Follow-Up Evaluation

After six months, 18 patients were reevaluated. Two patients were excluded for the development of major depression. Between the first and second followup, two patients experienced one clinical relapse each and were treated with high doses of intravenous methylprednisolone (one gram × 5 days) with complete recovery. Time elapsed from both relapses and the follow-up evaluation was longer than 30 days.

Compared to baseline, a significant favourable change was confirmed for fatigue symptoms reflected by the reduction in fatigue scales score: FSS (*P* = 0.0001), MFIS (*P* = 0.01), pMFIS (*P* = 0.025), and cMFIS (0.009). Furthermore, there was a trend for reduced psMFIS score (*P* = 0.054). Quality of life assessment showed a significant improvement in PHCS (*P* = 0.049) but not in MHCS (*P* = 0.30). No differences were detected for EDSS and MSFC scores (Table 2).

## 4. Discussion

Wrong strategies of movement planning, even if unconscious, may contribute to the development of fatigue in patients with MS. This hypothesis is mainly supported by functional neuroimaging studies, which suggested that fatigue in MS could result from altered connection between cortical and sub-cortical areas involved in motor planning [8–11].

 The premise of neurocognitive rehabilitation is to utilize a patient's strength to overcome weaknesses. Thus, the identification of individualized compensation strategies will result in less allocation of attention resources and less expanded effort, which is likely to favourably impact on fatigue severity and presence. Starting from this hypothesis, we tested the efficacy of a novel rehabilitation treatment which helps the patient to detect wrong strategies of motor planning and to learn more efficient movement execution, by using exercises based on MI, which basically is a process of mental simulation or rehearsal of actual movement. MI is a neurocognitive rehabilitation technique used in the treatment of chronic disorders other than MS (i.e., stroke), however, not specifically focusing on fatigue [26, 27]. MI is defined as a dynamic state, governed by the principles of central motor control, during which the representation of a specific motor action is internally reactivated within the working memory without any overt motor output [38, 39]. Previous studies demonstrated a close temporal coupling between MI and actual movement, that is, the time taken to mentally perform an action that closely mirrors the actual movement [38]. Furthermore, during imagined movement, the reduction in accuracy with increasing speed is maintained and the asymmetry between dominant and nondominant hand is also preserved [39]. Finally, functional studies have shown that MI and executed movements activate similar pathways [40], thus supporting the idea that MI is an integral part of the wider motor system that can be represented by internal models or programs, which develop over time and are consistently changing. 

The patients included in this study had no or only minimal disability (median EDSS score 2.0), nonetheless, any motor or cognitive task caused clinically relevant fatigue symptoms. We supposed a distortion of the representation of movement which could be correlated with the development of fatigue, even in patients where motor dysfunction is not clinically evident yet. The patient's verbal description of MI has been analysed to recognize the way she/he processed MI related to specific movements. This analysis enabled us to detect wrong effort strategies, that seem to be present already within MI, according to the presence of a “chaotic MI” which has been defined as an inability to perform MI accurately or as a temporal uncoupling typical of subjects with SNC lesions [26]. 

During exercise the physiotherapists guided the subjects to the correct perception of kinaesthetic information resulting in a more physiologic motor program that produced less or no fatigue and a better quality of executed movement, observed after the 5-week treatment. Our rehabilitation approach should lead the patients to learn new strategies of motor planning which might endure after treatment. 

In this sample of patients, the proposed treatment significantly reduced fatigue symptoms and improved to some extent also quality of life and disability. The results are particularly promising since the improvement was detectable also in the long-term followup, implying the acquisition of durable, more effective, motor strategies in these patients. In fact, the neurocognitive rehabilitation program showed a significant favourable impact on fatigue symptoms. This benefit persisted six months after the training. The treatment showed also a beneficial impact on quality of life and disability, but this effect disappeared on the 6-month followup, in line with the results of other rehabilitation therapies in MS [41]. The selective efficacy on fatigue symptoms six months after treatment is, in our opinion, an important result which suggests a focused effect of the proposed rehabilitation program. To maintain and apply the correct strategies of motor planning over time, a program of periodic retraining twice a year seems advisable. 

The present study has some methodological limitations, such as the lack of a control group and the small sample size. In further studies, a larger sample size and a comparison with a control group on another active treatment (i.e., aerobic exercise) would be advisable. Due to the lack of a control group, a partial placebo effect cannot be excluded, especially in fatigue, which can only be measured subjectively. Nonetheless, we believe that the results are interesting and warrant further studies to be confirmed, not only for the significant quantitative improvement in the fatigue scale scores but also for the qualitative amelioration of motor strategies attested by the physiotherapists at the end of the training and the persistence of the favourable effect on fatigue at the long-term followup, which is unlikely for a placebo effect. Although the PASAT test is a good marker of attention and speed processing, since the treatment requires a high level of attention, a thorough neuropsychological evaluation should be advisable before initiating the treatment

To expand research in this topic, an fMRI study in MS patients with and without fatigue and in healthy controls, with the aim to compare the patterns of activation in the cerebral cortex during a simple motor task, is under completion in our institution. It will be interesting in such a study to observe the changes of activation patterns after a training based on neurocognitive rehabilitation treatment to objectively confirm the modification of motor strategies in treated patients. 

In conclusion, our data suggest that neurocognitive rehabilitation based on MI could be an interesting approach for the management of fatigue symptoms in MS patients and that this strategy deserves further controlled studies to confirm its efficacy. Since in this sample of patients this treatment had a rapid effect, which tended to persist for some months, retraining may improve the efficacy in the longterm.

## Figures and Tables

**Table 1:** Mean clinical variable scores at baseline and after five weeks of treatment (*n* = 20).

Variable	Baseline mean (SD)	First follow-up mean (SD)	*P*
EDSS	2.45 (1.29)	2.48 (1.26)	N.S.

FSS	5.66 (0.48)	4.20 (1.07)	**<0.0001**

MFIS	36.50 (14.28)	23.80 (12.40)	**<0.0001**
pMFIS	20.15 (6.33)	13.45 (6.68)	**<0.0001**
cMFIS	14.15 (8.36)	8.55 (6.16)	**<0.0001**
psMFIS	2.20 (2.12)	1.70 (1.53)	0.133

MSFC	0.10 (0.61)	0.21 (0.56)	**0.035**
NHPT	22.04 (5.73)	21.04 (4.34)	0.083
25FWT	5.83 (1.78)	5.58 (1.94)	0.093
PASAT	39.55 (12.09)	41.65 (13.19)	0.095

MSQOL-54			
PHCS	52.46 (17.08)	65.17 (15.98)	**0.002**
MHCS	60.78 (16.80)	70.48 (16.33)	**0.004**

EDDS: Expanded Disability Status Scale, MSFC: Multiple Sclerosis Functional Composite, NHPT: Nine-Hole Peg Test, 25 FWT: 25-Foot Walking Test, PASAT: Paced Auditory Serial Addition Test, FSS: Fatigue Severity Scale, MFIS: Modified Fatigue Impact Scale, pMFIS: physical MFIS subscale, cMFIS: cognitive MFIS subscale, psMFIS: psychosocial MFIS subscale, MSQOL-54: Multiple Sclerosis Quality of Life Questionnaire, PHCS: Physical Health Composite Score, MHCS: Mental Health Composite Score.

**Table 2:** Mean clinical variables scores at baseline and 6 months after treatment (*n* = 18).

Variable	Baseline mean (SD)	Second follow-up mean (SD)	*P*
EDSS	2.50 (1.34)	2.53 (1.63)	N.S.

FSS	5.72 (0.47)	4.17 (1.31)	**<0.0001**

MFIS	36.67 (13.76)	26.78 (17.65)	**0.010**
pMFIS	20.28 (6.35)	15.71 (8.87)	**0.025**
cMFIS	14.33 (8.20)	9.72 (8.50)	**0.009**
psMFIS	2.06 (2.01)	1.33 (1.81)	0.054

MSFC	0.10 (0.63)	0.22 (0.64)	0.122
NHPT	22.21 (5.96)	20.97 (4.44)	0.220
25FWT	5.80 (1.83)	5.77 (2.57)	0.108
PASAT	39.56 (12.45)	41.89 (14.41)	0.216

MSQOL-54			
PHCS	52.80 (16.64)	60.78 (17.80)	**0.049**
MHCS	61.19 (15.56)	65.03 (18.48)	0.309

EDDS: Expanded Disability Status Scale, MSFC: Multiple Sclerosis Functional Composite, NHPT: Nine-Hole Peg Test, 25 FWT: 25-Foot Walking Test, PASAT: Paced Auditory Serial Addition Test, FSS: Fatigue Severity Scale, MFIS: Modified Fatigue Impact Scale, pMFIS: physical MFIS subscale, cMFIS: cognitive MFIS subscale, psMFIS: psychosocial MFIS subscale, MSQOL-54: Multiple Sclerosis Quality of Life Questionnaire, PHCS: Physical Health Composite Score, MHCS: Mental Health Composite Score.
